# Complete Genome Sequence of *Paenibacillus polymyxa* YC0136, a Plant Growth–Promoting Rhizobacterium Isolated from Tobacco Rhizosphere

**DOI:** 10.1128/genomeA.01635-16

**Published:** 2017-02-09

**Authors:** Hu Liu, Kai Liu, Yuhuan Li, Chengqiang Wang, Qihui Hou, Wenfeng Xu, Lingchao Fan, Jian Zhao, Jianyu Gou, Binghai Du, Yanqin Ding

**Affiliations:** a College of Life Sciences/Shandong Key Laboratory of Agricultural Microbiology/ National Engineering Laboratory for Efficient Utilization of Soil and Fertilizer Resources, Shandong Agricultural University, Taian, China; b College of Resources and Environment, Shandong Agricultural University, Taian, China; c State Key Laboratory of Nutrition Resources Integrated Utilization, Linshu, China; d Zunyi Tobacco Monopoly Administration of Guizhou, Zunyi, China

## Abstract

*Paenibacillus polymyxa* strain YC0136 is a plant growth–promoting rhizobacterium with antimicrobial activity, which was isolated from tobacco rhizosphere. Here, we report the complete genome sequence of *P. polymyxa* YC0136. Several genes with antifungal and antibacterial activity were discovered.

## GENOME ANNOUNCEMENT

*Paenibacillus polymyxa* is considered to be a plant growth–promoting rhizobacterium (1, 2). It can promote plant growth by multiple mechanisms, such as nitrogen fixation (3), production of indole-3-acetic acid (3, 4) and cytokinin (2), biosynthesis of siderophore (3), and inducing systemic resistance (5). *P. polymyxa* can inhibit the growth of plant pathogens by producing various antimicrobial substances, such as fusaricidin (6) and polymyxin (7). *P. polymyxa* YC0136 was isolated from the rhizosphere soil of tobacco in Guizhou, China. It presents antimicrobial activity against tobacco black shank caused by the soil-borne pathogen *Phytophthora parasitica* var. *nicotine*.

The complete genome sequencing of YC0136 was performed using the PacBio platform. A 8- to 10-kb DNA library was established by G-tubes. Genomic DNA was sequenced in a single-molecule real-time cell. Genomic DNA sequencing generated 136,809 reads and contained 1,087,154,601 bp. The largest read is 40,683 bp, and the sequencing coverage reached 193.0×. All reads were *de novo* assembled with HGAP version 2.3.0 (8). The genome sequence was annotated using the NCBI Prokaryotic Genome Automatic Annotation Pipeline (PGAP) (http://www.ncbi.nlm.nih.gov/genome/annotation_prok). We also analyzed the genome using the Carbohydrate-Active enZYmes Database (CAZy) version 20141020 (9) (http://www.cazy.org). Prophages were predicted with PHAST version 2013.03.20 (10) (http://phast.wishartlab.com), and antiSMASH version 3.0.5 (11) (http://antismash.secondarymetabolites.org) was used to analyze the secondary metabolism clusters.

*P. polymyxa* YC0136 consists of a 5,621,728-bp circle chromosome with 45.743% G+C content. A total of 4,847 genes were predicted, including 4,650 coding genes, 33 rRNAs, 92 tRNAs, four ncRNAs, and 68 pseudogenes. A total of 285 genes encoded carbohydrate enzymes, which comprised 135 glycoside hydrolases, 60 glycosyl transferases, 10 polysaccharide lyases, 44 carbohydrate esterases, seven auxiliary activities, and 29 carbohydrate-binding modules. Two prophages, of 19,197 bp and 20,348 bp, were discovered in YC0136. There were 14 gene clusters related to secondary metabolites, such as one polymyxin biosynthetic gene cluster (PPYC1_03440-03590), one tridecaptin biosynthetic gene cluster (PPYC1_12750-12910), and one fusaricidin biosynthetic gene cluster (PPYC1_23735-23930). Based on the comparative analysis of whole-genome sequences, *P. polymyxa* YC0136 showed a close phylogenetic relationship with *P. polymyxa* strain CR1.

### Accession number(s).

The chromosome sequence of* P. polymyxa* YC0136 has been deposited in GenBank under the GenBank accession number CP017967. The version described in this paper is the first version.
